# Addressing the need for standardization of test methods for self-healing concrete: an inter-laboratory study on concrete with macrocapsules

**DOI:** 10.1080/14686996.2020.1814117

**Published:** 2020-09-22

**Authors:** Tim Van Mullem, Giovanni Anglani, Marta Dudek, Hanne Vanoutrive, Girts Bumanis, Chrysoula Litina, Arkadiusz Kwiecień, Abir Al-Tabbaa, Diana Bajare, Teresa Stryszewska, Robby Caspeele, Kim Van Tittelboom, Tulliani Jean-Marc, Elke Gruyaert, Paola Antonaci, Nele De Belie

**Affiliations:** a Magnel-Vandepitte Laboratory, Department of Structural Engineering and Building Materials, Faculty of Engineering and Architecture, Ghent University, Ghent, Belgium; b Department of Structural, Geotechnical and Building Engineering, Politecnico di Torino, Turin, Italy; c Responsible Risk Resilience Centre, Politecnico di Torino, Turin, Italy; d Faculty of Civil Engineering, Cracow University of Technology, Cracow, Poland; e KU Leuven, Department of Civil Engineering, Materials and Constructions, Ghent Technology Campus, Ghent, Belgium; f Department of Building Materials and Products, Institute of Materials and Structures, Faculty of Civil Engineering, Riga Technical University, LV-Riga, Latvia; g Department of Engineering, University of Cambridge, Cambridge, UK; h INSTM Research Unit PoliTO-LINCE Laboratory, Department of Applied Science and Technology, Politecnico di Torino, Turin, Italy

**Keywords:** Round robin test, self-healing concrete, standardization, macrocapsules, polyurethane, capillary water absorption, water permeability, active crack width control technique, machine learning, 600 Others Self-healing concrete

## Abstract

Development and commercialization of self-healing concrete is hampered due to a lack of standardized test methods. Six inter-laboratory testing programs are being executed by the EU COST action SARCOS, each focusing on test methods for a specific self-healing technique. This paper reports on the comparison of tests for mortar and concrete specimens with polyurethane encapsulated in glass macrocapsules. First, the pre-cracking method was analysed: mortar specimens were cracked in a three-point bending test followed by an active crack width control technique to restrain the crack width up to a predefined value, while the concrete specimens were cracked in a three-point bending setup with a displacement-controlled loading system. Microscopic measurements showed that with the application of the active control technique almost all crack widths were within a narrow predefined range. Conversely, for the concrete specimens the variation on the crack width was higher. After pre-cracking, the self-healing effect was characterized via durability tests: the mortar specimens were tested in a water permeability test and the spread of the healing agent on the crack surfaces was determined, while the concrete specimens were subjected to two capillary water absorption tests, executed with a different type of waterproofing applied on the zone around the crack. The quality of the waterproofing was found to be important, as different results were obtained in each absorption test. For the permeability test, 4 out of 6 labs obtained a comparable flow rate for the reference specimens, yet all 6 labs obtained comparable sealing efficiencies, highlighting the potential for further standardization.

## Graphical abstract

**Figure:**
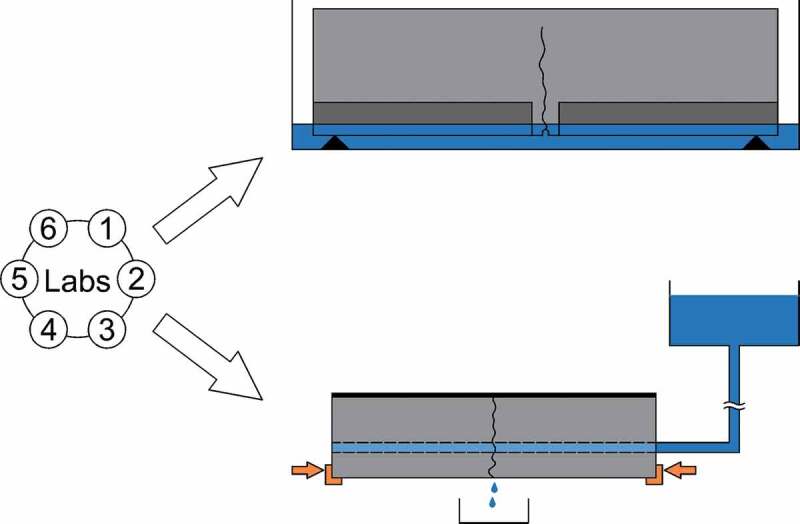

## 1. Introduction

Self-healing concrete has a great potential as a building material as it is able to heal its own defects without external human intervention. These defects are a common phenomenon in concrete and manifest as cracks caused by, e.g. mechanical loading or restrained shrinkage. In most cases, the formation of cracks does not pose an immediate risk for the structural behaviour of concrete elements. However, these cracks may significantly accelerate the degradation of the elements, and might thereby reduce the service life and the sustainability. To restore the damaged concrete, repair actions may need to be undertaken which are expensive due to the requirement of skilled labour and specialised repair products, on top of indirect costs such as temporary loss of function. By providing concrete with the ability to heal itself, through the addition of healing agents and changing the mix design, the initial construction cost requires an increased investment. Yet, the total lifetime cost can be reduced as a result of a decreased need for repair and maintenance, in addition to an extended service life. Structures which can operate longer without being replaced also have a significant environmental benefit, considering that the construction sector has a large share in the global CO_2_ emissions [1].

Many different self-healing methods for cementitious materials have been proposed [2]. A wide variety of test methods is already available to assess the performance of self-healing cementitious materials to be used in new structures [3] and to assess external surface treatments to repair existing structures [4]. Additionally, several numerical models investigating self-healing cementitious materials have been developed [5]. Nonetheless, it often remains difficult to compare results from different studies as no standard test methods are yet available to test the efficiency and the enhancement caused by the self-healing properties. This is made more difficult by the large variety of factors which can influence the self-healing behaviour [3]. This lack of standardized test methods for self-healing concrete hinders international collaboration and slows down further development. Additionally, it impedes commercialisation as it is difficult to convince the construction sector, which is used to a strictly regulated concrete production. In an effort to remediate this, six different inter-laboratory testing programs to evaluate test methods to assess the efficiency of self-healing concrete have been established within the framework of the EU COST Action CA 15,202 SARCOS [6]. In addition to the assessment of the test methods, the goal of these inter-laboratory testing programs is also to quantify the behaviour of the self-healing techniques in concrete instead of in cement paste or mortar which are often used in lab scale experiments. When upscaling from lab scale to real-life concrete mix designs, there is a dilution of the healing agents, when the dosage is kept constant with respect to the cement weight [2]. Increasing the dosage is often not desirable due to the negative effect on mechanical properties [2,7–12] and the increased cost. As a recognition to the versatility of self-healing cementitious materials, each of the six inter-laboratory testing programs focuses on a different self-healing technique: (1) concrete with mineral additions, (2) concrete with the addition of magnesium oxide, (3) concrete enhanced with crystalline admixtures, (4) high-performance fibre reinforced concrete enhanced with crystalline admixtures, (5) concrete with preplaced macrocapsules containing polymeric healing agent, and (6) concrete with encapsulated bacteria. The ongoing development of standardized test methods for self-healing concrete is ensured by the recent start of a large international Marie Curie training network called SMARTINCS (Smart, Multi-functional, Advanced Repair Technologies In Cementitious Systems), in which different self-healing methods and their commercialization potential will be further investigated [13,14].

This paper reports on the fifth inter-laboratory testing program, focussing on concrete with macrocapsules. The use of macrocapsules in cement composites already dates back to 90s [15–17]. Since then macrocapsules have been used to incorporate a wide variety of healing agents in cementitious materials [2,18–24]. The macrocapsules which were used in this study were glass tubular capsules filled with polyurethane. The glass capsules were placed in the moulds prior to casting so that their location was known and precisely controlled. This had the advantage that the healing agent had to be supplied only at the location of the specimen where cracks were expected, thus preventing the addition of healing agent whose healing potential would not be triggered. When the cementitious matrix cracks at the location of a capsule, the glass shell breaks, allowing the polymeric healing agent to flow out and seal the crack. Different studies have already shown good results for the same type of polyurethane encapsulated in glass capsules. For this healing mechanism, the bending strength can be partially regained (up to 35%) and once the polyurethane has hardened it is capable of bridging moving cracks (i.e. cracks with a changing width due to changes in the stress in the cross-section) with an additional crack opening between 50% and 100% [25]. The regain in liquid tightness (often referred to as sealing efficiency) is very good – even up to perfect – with regard to capillary water absorption [25–27] and water permeability [28]. In a preliminary study with regard to the recovery of the durability against carbonation, it was noted that more than half of the specimens behaved as if uncracked [29]. With regard to the resistance against chloride ingress, an accelerated chloride diffusion test showed a healing efficiency of 75% or higher in healed cracks from a depth of 6 mm onwards away from the exposed surface. In addition, a probabilistic service life prediction executed in the same study highlighted that for a reinforced concrete slab with this encapsulated healing agent in exposure class XS2 the first repair would only be needed after 60–94 years, instead of after 7 years [30]. Performing non-steady state chloride migration tests even showed a perfect durability recovery in all tested samples [31]. The good behaviour in chloride environments was also noted in a separate study which indicated an increased resistance with regard to chloride-induced reinforcement corrosion [32].

The inter-laboratory testing program was split up in two parts. In the first part, reinforced concrete specimens with and without capsules were cracked in a displacement-controlled three-point bending test (passive crack control). Subsequently, these concrete specimens were subjected to two capillary water absorption tests, each absorption test being executed with a different type of waterproofing applied on the zone around the crack. In the second part, unreinforced mortar specimens were cracked in a force-controlled three-point bending test after a Carbon Fibre Reinforced Polymer (CFRP) strip was glued on their top surface. Immediately after cracking, the crack width of the mortar specimens was reduced to the desired crack width using an active crack control technique [33]. Once the crack width of these prismatic mortar specimens was controlled, they were subjected to a water permeability test [10,34].

## 2. Materials and methods

This section provides information on the used healing agent, the specimen preparation, and the testing methods. In total, six university laboratories from five different European countries participated in this inter-laboratory testing program: Ghent University, Politecnico di Torino, Riga Technical University, Cracow University of Technology, Cambridge University, and KU Leuven (Ghent Technology Campus). All macrocapsules and all specimens were prepared at Ghent University. Cracking and subsequent testing were performed at the participating laboratories.

The inter-laboratory testing program which is reported here is the only one within the framework of the COST Action SARCOS which did part of the tests on mortar specimens. Unlike for the other healing techniques, the amount of polymeric healing agent is not bound to the mortar fraction, meaning that there is not necessarily a deficit of healing agent at the location of large aggregates, as might be the case for other healing agents. For macrocapsules the amount of healing agent is entirely determined by the amount of capsules, not by the mix design and the mortar/paste fraction. As long as the viscosity of the healing agent is within certain boundaries, the healing agent will fill the crack volume regardless of the amount of aggregates at the location of the crack. Boundaries of 100 to 500 mPa.s for the viscosity have been reported, as this is low enough to allow for an emptying of the capsules and a subsequent flow through the crack, but also high enough to prevent absorption of the agent by the matrix and to prevent the healing agent from seeping out of the crack [15]. Another reason for doing part of the tests on mortar instead of concrete, is that the studied healing mechanism has already been implemented in large concrete beams (150 x 250 × 3000 mm^3^) [35]. Analysing a test method on mortar specimens also allowed to accommodate an important part of the research field which is still in the prototype phase, during which healing agents are often expensive and screening on mortar is required prior to testing the efficiency in concrete.

### 2.1. Encapsulated healing agent

The polymeric healing agent which was used in this study was a liquid single-component polyurethane (PU) which is commercially available (HA Flex SLV AF, GCP Applied Technologies, Belgium). The PU had a low viscosity (<250 mPa.s at 25°C) and was developed as a resin for the manual injection of small cracks (<0.5 mm). Upon contact with moisture, which is present in the air or in the concrete matrix, the PU polymerizes. When the product comes into contact with liquid water it can undergo an expansive foaming reaction, as a result of water reacting with the isocyanate groups causing the formation of carbon dioxide. To protect the agent from polymerizing up until the moment of crack initiation, the agent was encapsulated in tubular capsules. These macrocapsules were made from borosilicate glass with an external, respectively, internal, diameter of 3.35 mm and 3 mm (Hilgenberg, Germany). Glass macro-capsules are considered to be representative also for other types of brittle capsules, e.g. capsules made from cement paste [24,36–38], ceramic material [39–41] or PMMA [42,43]. The length of the capsules varied depending on the type of specimen. Yet, the ratio of the total volume of healing agent (calculated as the sum of the internal volume of all capsules in a specimen) over the theoretical crack volume (approximated as a triangular shaped bending crack varying linearly from the bottom of the specimens to the top) was kept constant. To estimate the volume of healing agent in a capsule, an effective length of the capsule was assumed, being 5 mm shorter than the total length. This was done to account for a small amount of air in the capsule, as well as the sealing of the capsules with a two-component epoxy glue (PC 5800, Tradecc, Belgium).

To manufacture the macrocapsules, one side of the tubular glass capsules was first sealed with epoxy glue which was allowed to cure overnight. Subsequently, the capsules were filled with the PU healing agent using a syringe with needle. Care was taken to limit the amount of entrained air. The used PU had a weak yellow colour. To make the leakage of PU from the cracked specimens more visible, a small amount of bright yellow fluorescent powder dye (EpoDye, Struers, the Netherlands) was mixed into the PU prior to filling the capsules. After filling the capsules with PU, the open side of the capsules was also sealed using epoxy glue.

Specimens which contained capsules were denoted as CAPS specimens, as opposed to reference specimens without capsules which were denoted as REF specimens. Both the REF and the CAPS specimens were cracked. Part of the concrete reference specimens without capsules remained uncracked to determine the sorption coefficient on uncracked concrete, these were denoted as UNCR specimens.

### 2.2. Concrete specimens

#### 2.2.1. Concrete specimens preparation

Concrete prisms with a dimension of 60 × 60 x 220 mm^3^ were cast using the mix composition given in Table 1 (an average air content of 2% was assumed in the calculation). The dimensions were chosen to allow for an easy handling during the capillary water absorption test (see section 2.2.3) and to optimize shipping. As a result of these dimensions the maximum aggregate size was limited to 8 mm. The used cement was a CEM I 42.5 N (ENCI, the Netherlands) and the water to cement ratio was equal to 0.55. To improve the workability and to allow the concrete to easily flow around the macrocapsules, 0.41 m% of a superplasticizer based on modified polycarboxylic ether polymers (Master Glenium 27 concentration of 20%, BASF, Belgium) was added relative to the weight of cement. The dry components were first mixed for 1 min (vertical shaft mixer with rotating pan and capacity of 50 litres, Eirich, Germany), after which the water together with the superplasticizer were added and the mixing operation continued for another 2 min. Due to the large quantity of specimens which needed to be cast (12 REF prisms, 12 CAPS prisms and 3 UNCR prisms for each lab), individual batches were prepared for each lab.

**Table 1.** Concrete mix composition.

Components	Amount
Cement CEM I 42.5 N	337.6 kg/m^3^
Water	185.2 kg/m^3^
Sand (0–5 mm)	742.9 kg/m^3^
Gravel (2–8 mm)	1013.1 kg/m^3^
Limestone filler	58.0 kg/m^3^
Superplasticizer Master Glenium 27	1328 ml/m^3^

**Figure 1.**
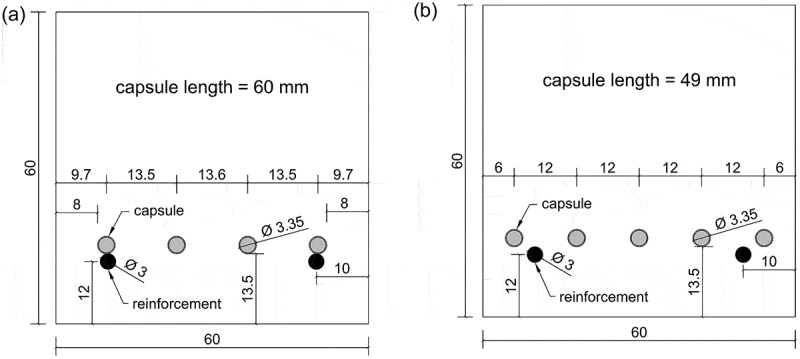
Cross-section of concrete specimens (a: layout with four capsules, b: layout with five capsules) (dimensions in mm).

**Figure 2.**
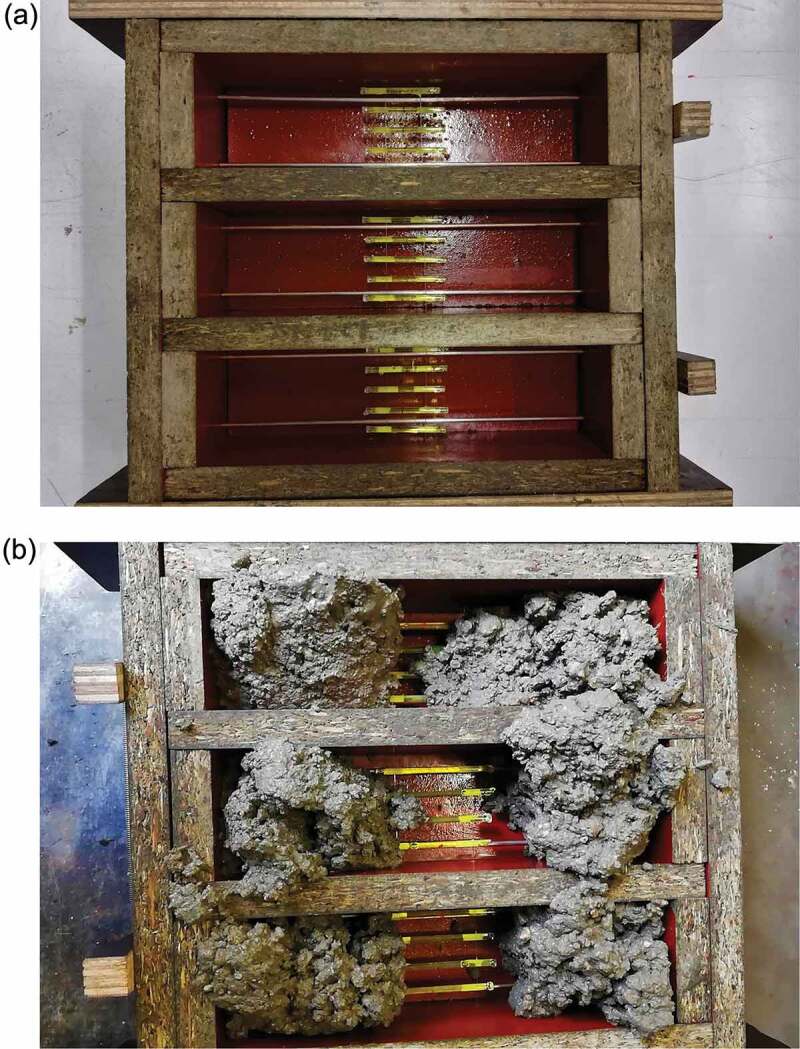
Moulds to make concrete specimens (60 × 60 × 220 mm^3^) with capsules (a: mould with five capsules; b: casting of specimens with four capsules).

Two smooth reinforcement bars with a diameter Ø of 3 mm (low-alloyed steel TIG welding rods, Hilco, Germany) were positioned at 12 mm from the bottom of the specimens, see Figure 1. To improve the bond between the bars and the concrete matrix, they were slightly manually roughened using sandpaper. All labs received REF specimens without macrocapsules. For the CAPS specimens two different layouts were considered. Lab 1 to 5 received concrete specimens with four macrocapsules with a length of 60 mm, see Figure 1a. Lab 6 received concrete specimens with five macrocapsules with a length of 49 mm, see Figure 1b. By changing the capsule length, the total volume of healing agent was the same in the two layouts. The position of the capsules was determined by assuming an equal outflow radius out of each capsule, i.e. assuming an identical circular outflow. For the layout with four capsules, the two outermost capsules (i.e. the capsules closest to the side faces of the mould) were positioned so that the largest aggregates could pass between the capsule and the side faces of the mould, which was done out of fear for compaction errors. As a consequence, the outer capsules in that layout needed to supply healing agent to a slightly larger area of the crack in order to obtain perfect healing, see Figure 1 a. For the layout with five capsules, the requirement that the largest aggregates should be able to pass between the capsules and the side faces was not imposed as this would cause too much deviation from the identical circular outflow for the outermost capsules. Instead extra care was taken in filling and compacting these moulds. In both layouts, capsules were prepositioned in the moulds by gluing them on a nylon wire running just above the reinforcement, see Figure 2.

All specimens were compacted on a vibration table. For the specimens with capsules, concrete was first placed next to the capsules, see Figure 2. The vibration table was then activated allowing the concrete to flow under and between the capsules, after which the rest of the concrete was added. The specimens were stored in a curing room (20°C and >95% relative humidity RH) and the day after casting they were demoulded. No compaction defects were visible for the specimens with five capsules, despite the limited distance between the outer capsules and the walls of the mould. The specimens were sealed in plastic foil in groups of three to prepare them for shipping. Up until the moment of shipping the specimens remained in a climate room at 20°C.

For each batch of specimens, the workability (determined by flow table testing according to EN 12350–5), the fresh density (EN 12350–6) and the air content (pressure gauge method following EN 12350–7) were determined. Additionally, a minimum of three control cubes with a side of 100 mm were taken from all batches to determine the concrete compressive strength (EN 12390–3). The cubes were demoulded at the same time as the test specimens and were sealed in plastic foil in the same curing room at 20°C. The strength testing for all specimens was performed at Ghent University.

#### 2.2.2. Concrete specimens cracking

Prior to cracking the concrete specimens, the different participating labs sawed a notch with a depth of 3 ± 2 mm in the bottom of the specimens at the middle of the span. The average depth of the notch $d_{notch}$ for the different labs is given in Table 2. At an age of 15 ± 1 days after casting the specimens were cracked in a three-point bending test with a span of 190 mm. Depending on the lab, the crack formation was controlled using a displacement-controlled loading system by means of either a linear variable differential transformer (LVDT) or a crack mouth opening displacement (CMOD) clip gauge mounted on the bottom of the specimens. One lab used digital image correlation (DIC) measurements to control the crack formation. The crack was opened at a speed of approximately 0.7 µm/s. The target crack width at the crack mouth after unloading was 300 µm. To achieve this, several labs opened the cracks to a wider extent to account for an elastic closure of the crack upon removal of the load as a result of an elastic shortening in the reinforcement. Table 2 provides an overview of the used displacement-controlled loading system, as well as the maximum crack width $w_{max}$ measured by the system prior to unloading. It should be noted that lab 1 and 6 used exactly the same test machine, displacement-controlled loading system, and test procedure, although the operator was different.

**Table 2.** Displacement-controlled loading systems, ultimate crack widths during loading w_max_ (after which specimens were unloaded) and average notch depth d_notch_ of the different labs.

Lab	Loading systems	*w*_max_ (µm)	*d*_notch_ (mm)
1	LVDT	485	3.5
2	CMOD	485	4.3
3	DIC	300	4.0
4	CMOD	300	1.5
5	CMOD	400	4.5
6	LVDT	485	4.3

After cracking of the specimens, they were stored in a dry lab environment with their crack facing downwards for minimally 24 hours to allow the PU to polymerize inside the crack. Afterwards, specimens were submersed in demineralized water for 24 hours, to make sure all the PU had polymerised. Prior to submersion, the crack width of the specimens was measured.

#### 2.2.3. Capillary water absorption to assess sealing efficiency of concrete specimens

Prior to the capillary water absorption test, the specimens were dried in an oven at 40°C for a minimum of 14 days until constant weight was achieved. Constant weight was considered to be achieved when the change in mass over a period of 2 hours was less than 0.2%. The specimens were subsequently acclimatized for 1 day at 20°C and 60% RH. Prior to testing, the specimens were partially waterproofed using adhesive aluminium tape to avoid that absorption through the matrix could be dominant with respect to absorption through the crack. The bottom and side of the specimens were waterproofed except for a zone on the bottom with a width of 14 mm centred around the crack. This to allow for possible deviations of the crack path from the notch and to cover small damages on the bottom surface of the specimens due to the removal of the attachments glued on the specimens to secure the CMOD or LVDT system during cracking.

The dry weight of the specimens (with waterproofing) was recorded and subsequently the specimens were placed in contact with water. The specimens were placed on spacers so that there was a volume of water under the specimen. The water level in the containers was approximately 3 ± 1 mm above the top of the notch. During a period of 24 hours, the specimens were one by one taken out of the water at predefined time steps (after 10 min, 20 min, 30 min, 1 h, 1 h 30 min, 2 h, 3 h, 4 h, 6 h, 8 h, and 24 h). The excess water on the surface was removed using a slightly pre-wetted cloth and the weight of the specimen was recorded, after which the specimen was immediately placed back in the water and the next specimen was taken out.

The result was plotted in a graph (x-axis: √time (√h), y-axis: water uptake (g)). The slope of the line was termed the sorption coefficient *SC* The sealing efficiency SE_abs_ was calculated as:

(1) $$SE_{abs.}=\frac{{\bar{SC}}_{REF}-{\bar{SC}}_{CAPS}}{{\bar{SC}}_{REF}-{\bar{SC}}_{UNCR}}$$

with:

- ${\bar{SC}}_{REF}$ the average sorption coefficient of the cracked reference specimens (g/√h);

- ${\bar{SC}}_{CAPS}$ the average sorption coefficient of the cracked self-healing specimens containing capsules (g/√h);

- ${\bar{SC}}_{UNCR}$ the average sorption coefficient of the uncracked reference specimens (g/√h).

To investigate the influence of the sealing quality, the aluminium tape was removed from the specimens and the specimens were stored again for 14 days in an oven at 40°C. They were then taken out of the oven for 3 days to apply a water impermeable coating (depending on the lab either an epoxy or a polyurethane was used). After the coating had dried, specimens were moved back into the oven for 3–4 days. Next, they were acclimatized for 1 day at 20°C and 60% RH and the capillary water absorption test was repeated.

An instruction video of the capillary water absorption test can be found in the supplementary material.

### 2.3. Mortar specimens

#### 2.3.1. Mortar specimens preparation

Unreinforced mortar prisms (40 x 40 x 160 mm^3^) were cast using the mortar mix composition given in Table 3 (an average air content of 4.5% was assumed in the calculation). The same cement and sand were used as for the concrete samples; however, the sand was sieved so that the maximum aggregate size was 2 mm. The water to cement ratio was 0.50. Superplasticizer based on modified polycarboxylic ether polymers (Master Glenium 27 concentration of 20%, BASF, Belgium) was added at a dosage of 0.16 m% relative to the weight of cement. The dry components were first mixed for 1 min (forced action pan mixer with a maximum capacity of 14 litres, CreteAngle, UK), after which the water together with the superplasticizer were added and the mixing operation continued for another minute. Any mortar sticking to the sides of the mixing bowl was manually scraped off and the mortar was mixed for an additional minute. For each lab a separate batch was made to cast the specimens, similar as for the concrete specimens (see section 2.2.1).

**Table 3.** Mortar mix composition.

Components	Amount
Cement CEM I 42.5 N	519.0 kg/m^3^
Water	257.7 kg/m^3^
Sand (0–2 mm)	1313.1 kg/m^3^
Limestone filler	89.0 kg/m^3^
Superplasticizer Master Glenium 27	787 ml/m^3^

**Figure 3.**
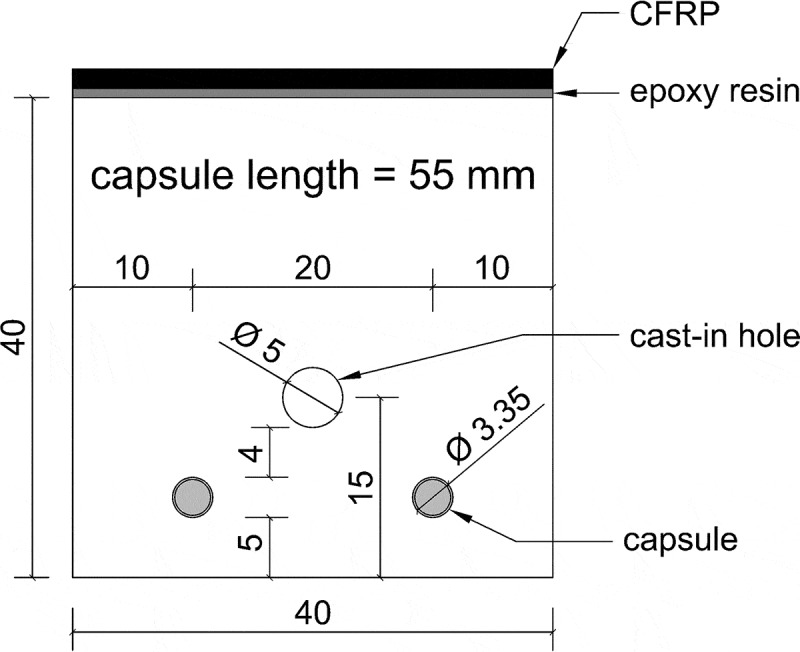
Cross-section of mortar specimens with capsules (dimensions in mm).

The mortar prisms remained unreinforced. Yet, the specimens were provided with a cast-in hole in order to perform the water flow test (see section 2.3.3). This cast-in hole was created by placing a smooth steel bar (Ø 5 mm) along the length of the moulds. This smooth steel bar was covered with demoulding oil. When the specimens were demoulded, the day after casting, the steel bar was pulled out of the mortar prisms, creating a hollow core along the longitudinal axis of the specimens. The cast-in hole was located with its centre at 15 mm from the bottom side of the specimens, see Figure 3. For the self-healing specimens the capsules were placed at a height of 5 mm above the bottom side of the specimen so that the vertical distance between the cast-in hole and the capsule, and the distance between the capsule and the bottom side of the specimen was approximately equal. The moulds were filled in two layers and every layer was compacted on a jolting table by 60 jolts (in accordance with EN 196–1). The mortar specimens were stored in a curing room (20°C and >95% RH) and the day after casting they were demoulded. After demoulding, the specimens were sealed in plastic foil in groups of three to prepare them for shipping. Up until the moment of shipping the specimens remained in a climate room at 20°C.

Aside from the test specimens, three prisms were made from each batch to determine the strength at 14 days according to the method described in EN 196–1. The strength testing for all specimens was executed at Ghent University. For each batch, the workability (according to EN 1015–3, table was not lubricated with oil but with a damp cloth), the fresh bulk density (according to EN 1015–6) and the air content (according to EN 1015–7) were determined once. To determine the fresh bulk density and the air content, the measuring vessel was filled in two layers which were each compacted by 60 jolts on a jolting table, similar to the test specimens.

#### 2.3.2. Mortar specimens cracking and active crack width control

**Figure 4.**
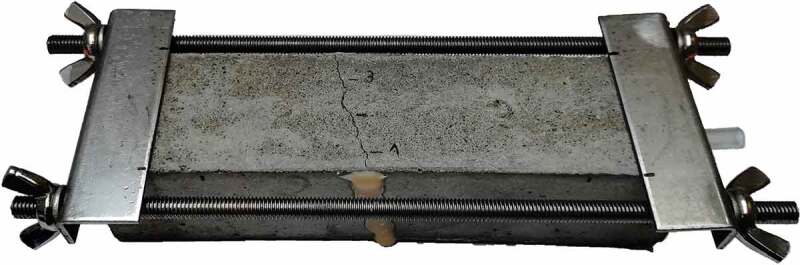
Screw jacks used for actively restraining the crack width of a mortar specimen.

Prior to shipping the mortar specimens, a carbon fibre reinforced polymer (CFRP) strip (PC® Carbocomp UNI, Tradecc, Belgium) with dimensions of 40 × 160 mm^2^ was glued on the top of the specimens using an epoxy resin (Sikadur®-30, Sika, Belgium), see Figure 3. Due to the limited dimensions, the specimens were not provided with a notch. On the one hand this had the disadvantage that the crack path was a bit more tortuous, on the other hand the crack pattern was more natural. At an age of 14 or 15 days, each of the different labs cracked the specimens in a three-point bending test with a span of 100 mm and a loading rate of 50 N/s (similar to the bending test prescribed by EN 196–1). The specimens were positioned so that the CFRP strip was at the top (i.e. the compression side). Due to the fact that there was no tensile reinforcement in the specimens they failed suddenly; however, both halves remained connected due to the CFRP strip. Due to the stiffness of the CFRP there was only one degree of freedom; the cracks could widen or close [33]. Immediately after cracking, the specimens were placed with their crack mouth facing upwards and the crack width was restrained using screw jacks (shipped by Ghent University) to approximately 400 µm, see Figure 4. The crack width was then further restrained under an optical microscope using an iterative procedure of measuring and restraining until the average crack width (see section 2.4) fell within the desired crack width range of 290 to 310 µm. The specimens were then turned so that the crack mouth was facing downwards (i.e. CFRP facing upwards). In order to limit the influence of the specimen orientation on the outflow of PU from the capsules, specimens (both from the CAPS series and the REF series) were cracked and immediately restrained prior to cracking a new specimen. The entire process of restraining and measuring the crack width of a specimen was executed in less than 30 min.

#### 2.3.3. Water permeability test to assess sealing efficiency of mortar specimens

**Figure 5.**
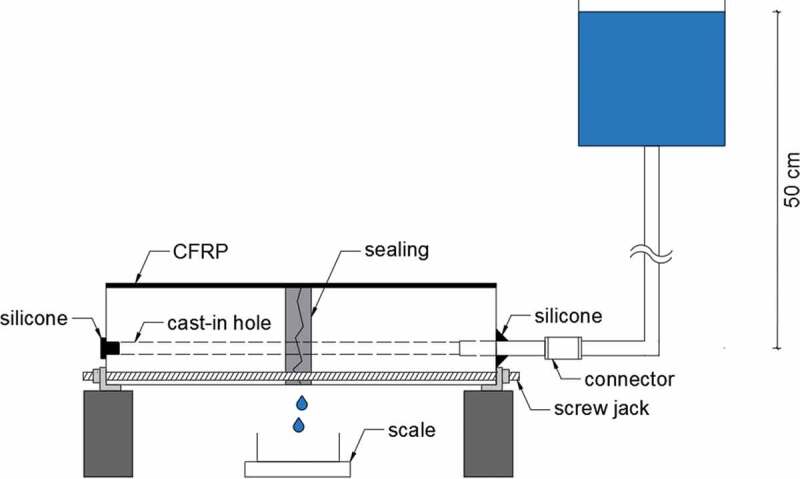
Water permeability measured by a constant head water flow test [33].

To measure the water permeability of the specimens a water flow test was used [10,33,34,44,45]. Prior to executing the test, specimens were stored dry in an indoor climate with their crack facing downwards for at least 1 day to allow the PU to polymerize. Afterwards, specimens were submersed in demineralized water for 24 to 48 hours to prevent any influence on the results from water absorption by the matrix. The specimens were then taken out of the water and were surface dried. To connect the specimens to the water flow setup, the cast-in hole was enlarged on one side to a diameter of 6 mm over a length of 25 ± 5 mm using a drill. This was done prior to cracking to prevent the vibrations from influencing the crack. A short tube (length of ± 60 mm, Ø_external_ 6 mm, Ø_internal_ 4 mm) was then inserted in the cast-in hole and a watertight connection was ensured using silicone. The other side of the cast-in hole was sealed completely (e.g. with silicone or a rubber stopper), see Figure 5. The inserted tube was connected to a tube (length of 130 ± 10 cm, Ø_external_ 6 mm, Ø_internal_ 4 mm) in contact with an open water reservoir. Instead of drilling and inserting a short tube, lab 4 used a plastic sleeve with a changing diameter so that on one side it could fit inside the cast-in hole and on the other side it could slide over the tube which connected with the water reservoir. The water head, measured from the cast-in hole in the specimens up to the water level, was kept constant throughout the test at 50 ± 2 cm by topping up with demineralized water whenever required. Water from the reservoir flowed through the tubes into the cast-in hole, from where it could leak out of the specimens via the crack. Only the water leaking out of the crack mouth, i.e. the bottom side of the specimens, was considered. Therefore, the sides of the specimens were sealed prior to saturation by using aluminium tape, silicon sealant, or a viscous glue (e.g. viscous methyl methacrylate, see Figure 4). The first 60 s that water was leaking from the crack were not recorded in order to measure only a fully developed flow and to allow water bubbles to be flushed from the system. Subsequently, the weight of the water which leaked from the crack was recorded for a minimum duration of 6 min.

The sealing efficiency $SE_{flow}$ of CAPS specimens with respect to REF specimens was calculated as:

(2) $$SE_{flow}=\frac{{\overset{ˉ}{q}}_{REF}-{\overset{ˉ}{q}}_{CAPS}}{{\overset{ˉ}{q}}_{REF}}$$

with: ${\overset{ˉ}{q}}_{REF}$ the mean water flow (g/min) of the reference specimens and the mean water flow ${\overset{ˉ}{q}}_{CAPS}$ (g/min) of the self-healing specimens containing capsules.

The employed water permeability test has already been investigated in a previous round robin test assessing the sealing efficiency of mortar with the addition of a bacteria based self-healing agent [44]. Unfortunately, this study was not able to come to conclusive results which was partly attributed to the large variation of the crack widths between different labs, as well as within individual labs. Additionally, it was argued that the cast-in hole was positioned too high in the specimens. A more recent study, in which the cast-in hole was positioned lower in the specimens and an active crack width control technique was applied to reduce the variation on the crack width, showed that it is possible to come to consistent results with this water permeability test [33].

#### 2.3.4. Visual examination of healing agent on the crack surfaces of mortar specimens

After performing the water permeability test, the CAPS specimens were split at the location of the crack to determine the healing agent coverage [25–27,36,37]. Pictures were taken from both crack faces of each specimen. The PU spread was determined via machine learning by using the Trainable Weka Segmentation plugin which is part of the open source software ImageJ (Fiji version 1.52) [46]. After manually training the machine learning algorithm, it was possible to produce a pixel-based segmentation of the zones with and without PU. The segmented images were then filtered to remove outliers, after which the images were manually checked for misidentified zones, e.g. a sand particle being identified as PU due to similar colour. The application of the Trainable Weka Segmentation to analyse images has also been used by Rodríguez et al. [47] to segment swollen SAP particles in tomography images of cracked mortar. Once the images were manually checked, the area with and without PU was determined. The surface coverage is the ratio of the area with PU over the total area (including cast-in hole) and is in this paper reported as the average from both crack faces of a specimen, as it was noted that the PU spread on the segmented images was similar on each crack face. This is different from a study by Van Belleghem et al. [26] who worked with the same PU and noted that the PU fractured at the contact surface with the mortar and was thus only visible on one of the crack surfaces. In the current study, fluorescent powder dye was added to the PU to make it more discernible. This dye left an imprint on the crack surfaces.

### 2.4. Crack width measurement

After cracking, the crack width at the crack mouth was determined using an optical microscope. Along the crack path different locations (three for mortar specimens and four for concrete specimens) were chosen to measure the crack width. The operator chose locations representative for the crack. Thus, the locations were not fixed as this would pose the risk of studying a location with a defect, e.g.: a missing aggregate or sand particle, (semi) loose particles, missing pieces of the cementitious matrix or parallel cracks. In order to standardise the crack width measurement as much as possible, the operators were provided with guidelines, see supplementary material. Images taken at locations with a defect would have resulted in the measurement of a local phenomenon, instead of a desired global description of the crack. In each location the crack width was measured 5 times. The reported average crack width was calculated as the average of the dataset compiled from all measuring points over the different locations of a crack. This means that, e.g. for a mortar prism the average was calculated from 15 points, i.e. 5 points for each of the three locations, representative for the crack.

## 3. Results and discussions

### 3.1. Concrete specimens

#### 3.1.1. Fresh and hardened properties of concrete

For each lab a separate batch of concrete was prepared at Ghent University. For each individual batch the workability (by flow table test), fresh density and air content were determined, see Table 4. The workability of all batches was comparable, with the batch used to produce specimens for lab 4 having a slightly lower workability and the batch used to produce specimens for lab 6 having a slightly higher workability. The air content was relatively constant and varied from 1.6% to 2.6%.

**Table 4.** Fresh (flow, fresh density, air content) and hardened properties (hardened density and compressive strength) of the different concrete batches used to cast the specimens of each lab (µ = mean, CoV = coefficient of variation, NA = not available).

					Hardened density (kg/m^3^)		Compressive strength (MPa)	
	Flow (cm)	Fresh density (kg/m^3^)	Air content (%)	Age (days)	*µ*	CoV	*µ*	CoV
Lab 1	46.5	2315	2.6	15	2328	0.1%	42.4	2.6%
Lab 2	47.5	2331	1.7	14	2318	0.2%	39.8	3.6%
Lab 3	46.5	2350	2.0	14	2334	0.2%	41.2	0.8%
Lab 4	44.3	2334	1.6	28	2320	0.5%	41.5	4.2%
Lab 5	NA	NA	NA	5	2289	0.7%	30.1	6.2%
Lab 6	49.8	2313	2.2	14	2345	0.2%	46.7	3.5%

For each individual batch the compressive strength (along with the hardened density) was also determined at Ghent University on cubes with a side of 100 mm. The age at testing of the different batches is given in Table 4. For four batches, tests were executed at 14 (+1) days. The cubes made from the batch used to cast the specimens of lab 4 were tested at 4 weeks to determine the 28 day compressive strength, and the cubes made from the batch used to cast the specimens of lab 5 were tested at 5 days, which approximately corresponded to the time that most specimens were shipped. From these results, it is clear that specimens had obtained approximately 70% of their compressive strength in the first week. After 2 weeks, the concrete (nearly) obtained its full compressive strength. It is noted that the compressive strength of the concrete of batch 6 was slightly higher than that of the other labs.

#### 3.1.2. Cracking and crack width of concrete specimens

**Figure 6.**
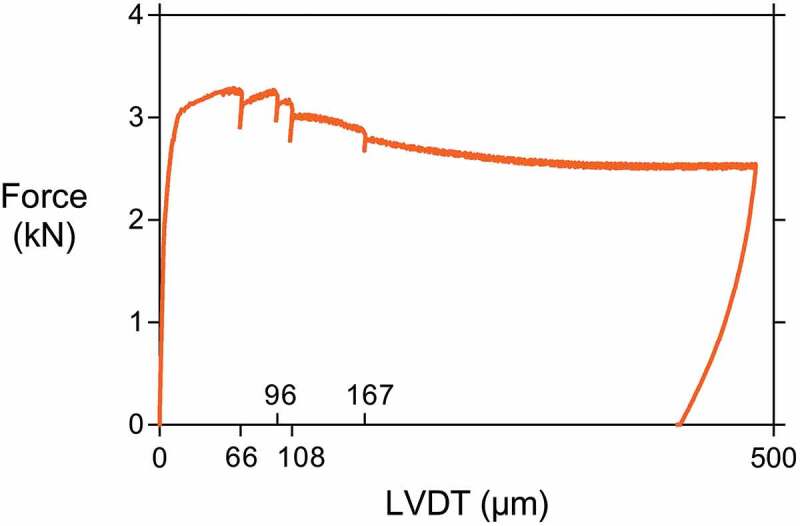
Load displacement graph of a CAPS specimen showing capsule rupture at 66, 96, 108, and 167 µm.

From the data recorded during crack creation by three-point bending, controlled by using a displacement-controlled loading system, it was possible to plot load-displacement graphs. In the case of CAPS specimens, small drops in the load indicated the rupture of capsules. Figure 6 shows the load displacement graph of a representative concrete specimen with four capsules. In the represented case the four capsules broke at: 66, 96, 108 and 167 µm of crack mouth opening. For some specimens, it was not possible to discern a discrete load drop for all of the contained capsules. The reason for this could be noise on the data or simultaneous breaking of multiple capsules. It has been reported for the type of capsules used in this study that the crack width at rupture at the location of the capsule is approximately 25 µm [48]. This value was surpassed in all specimens; therefore, it can be assumed that all capsules inside of the concrete specimens ruptured.

**Figure 7.**
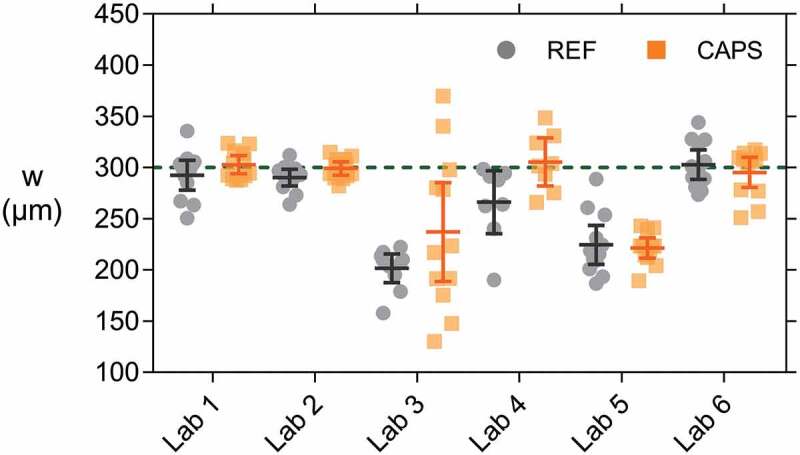
Crack width w of individual concrete specimens for which the mean of the series are indicated by horizontal lines and error bars give the 95% confidence interval on this mean. The horizontal dashed line indicates the target crack width of 300 µm.

After cracking, the crack width *w* at the bottom side of the concrete specimens was measured. Figure 7 shows the individual mean crack width of each specimen, as well as the mean of the series (horizontal lines) and the 95% confidence interval on this mean (error bars) for both REF and CAPS specimens. The horizontal-dashed line indicates the target crack width of 300 µm. It is evident that there was quite some variation on the results, even though two minimal outliers (one of lab 3 REF and one of lab 4 REF) and two maximal outliers (two of lab 4 CAPS) were discarded from the dataset prior to plotting this graph and performing the subsequent statistical analysis. Table 5 shows the number of specimens for each lab after removing the outliers (lab 3 and lab 4 also lost some specimens while calibrating the cracking procedure). For each lab it was statistically analysed if the mean crack width of the REF, respectively CAPS specimens, was equal to the target crack width of 300 µm (level of significance = 5.0%). Table 6 indicates that this hypothesis was not valid in the case of: the REF series of lab 2, both the REF and CAPS series of lab 3 and 5, and the REF series of lab 4. For the REF series of lab 2, the difference was rather moderate; the mean was practically equal to the target with a mean value of 290 µm, but the statistical test indicated a significant difference from 300 µm due to the low variation on the results, see also Figure 7. For each lab it was also analysed using independent sample t-tests (level of significance = 5.0%) if the mean crack width of the REF series was equal to the CAPS series. Table 6 indicates that only for lab 4 a moderately significant result was obtained (probability value *p* = 3.0%). Indeed, Figure 7 shows that the CAPS specimens of lab 4 had a slightly wider crack width than the REF specimens. To study if there was a significant difference for the crack widths obtained by the different labs the results of the REF and CAPS specimens were taken together – despite the difference between the REF and CAPS series of lab 4. Equal variances could not be assumed in the analysis (level of significance = 5.0%, *p* ≈ 0%). Therefore, the equality of means was investigated by both a Welch test and a Brown–Forsythe test. Both tests showed that the means were not all equal (level of significance = 5.0%, *p* ≈ 0%). In the post hoc analysis, a Tamhane’s T2 test revealed two separate groups: the means of lab 1, 2, 4 and 6 were equal (level of significance = 5.0%, *p* > 97.0%) and the means of lab 3 and 5 were equal (level of significance = 5.0%, *p* ≈ 100%).

**Table 5.** Number of concrete specimens taken into account for the analysis of the crack width and the capillary water absorption (NA = not available). The numbers between brackets for lab 6 are the number of specimens used for the repeated test with a new aluminium tape waterproofing.

	Crack width			Capillary water absorption test					
				Aluminium tape			Coating		
	REF	CAPS	UNCR	REF	CAPS	UNCR	REF	CAPS	UNCR
Lab 1	12	12	3	12	12	3	7	9	3
Lab 2	12	12	3	12	12	3	12	12	3
Lab 3	10	12	3	9	12	3	NA	NA	NA
Lab 4	8	8	3	8	8	3	8	8	3
Lab 5	12	12	3	12	12	3	12	12	3
Lab 6	12	12	3	12 (6)	12 (6)	3 (3)	12	12	3

**Table 6.** Mean *µ* and coefficient of variation CoV for the measured crack width *w*, as well as the probability value (*p*-value) for the statistical test comparing the mean to the target crack width of 300 µm and the *p*-value for the test comparing the mean of the REF to the mean of the CAPS.

		*w*		*p*-value	*p*-value
		*µ* (µm)	CoV	hypothesis *µ* = 300 µm	hypothesis *w_REF_* = *w_CAPS_*
Lab 1	REF	292	7.9%	28.4%	19.9%
	CAPS	303	4.6%	50.1%	
Lab 2	REF	290	4.4%	2.3%	7.5%
	CAPS	299	3.4%	76.3%	
Lab 3	REF	202	9.7%	0.0%	14.5%
	CAPS	237	32.1%	1.5%	
Lab 4	REF	266	13.8%	3.5%	3.0%
	CAPS	305	9.2%	60.2%	
Lab 5	REF	224	13.3%	0.0%	74.5%
	CAPS	221	7.0%	0.0%	
Lab 6	REF	303	7.5%	67.7%	42.7%
	CAPS	295	7.9%	49.1%

It is surprising that lab 4 obtained wider crack widths than lab 5 (especially for the CAPS specimens) since they both used a CMOD for controlling the cracking process and the ultimate crack opening under load was higher for lab 5 than for lab 4 (400 versus 300 µm, see Table 2). This might be partially explained by the fact that the notch was, on average, sawn less deep in the specimens of lab 4 compared to the specimens of lab 5 (1.5 mm compared to 4.5 mm, see Table 2). As a result, the microscopic measurements of the crack mouth (i.e. inside the notch) of the specimens of lab 4 turned out to be closer to the CMOD values recorded under load. From Table 6 it can also be noticed that the variation on the crack width of the CAPS specimens tested by lab 3 is very large (coefficient of variation CoV = 32.1%). Possibly the capsules induced an unsteady behaviour which made crack control via DIC not ideal. The low crack width of the REF specimens of lab 3 can be explained by the final crack opening which was only 300 µm and was measured by the DIC system directly in the notch. Upon load removal, the steel reinforcement underwent some elastic regain thereby partially closing the crack. The fact that lab 1, 2 and 6 obtained the target crack width of 300 µm was not entirely surprising; they opened the cracks the widest, up to a maximum value recorded by the loading system equal to 485 µm, see Table 2. The opening of the crack was controlled by a CMOD or LVDT positioned at the bottom of the specimens. At the location of the crack mouth (inside the notch) the crack width was a little bit lower but still larger than 300 µm. Upon removal of the load, the steel reinforcement underwent some elastic regain, closing the crack a little bit, thereby obtaining the target crack width of 300 µm.

#### 3.1.3. Capillary water absorption of concrete specimens

**Figure 8.**
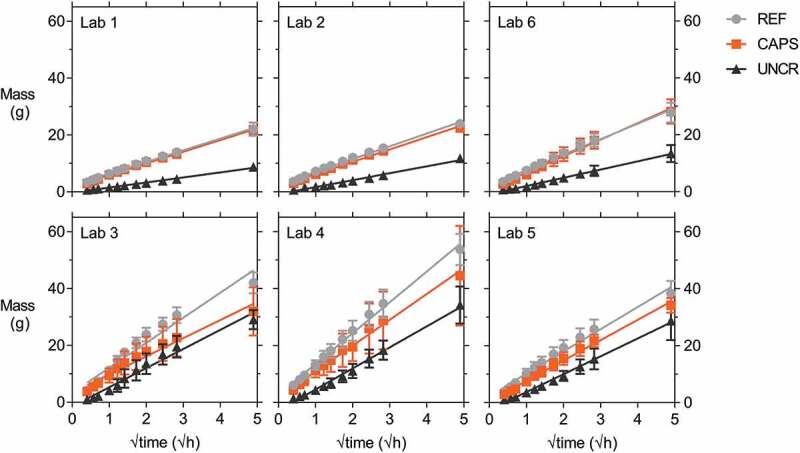
Cumulative mass gain versus the square root of time of REF, CAPS and UNCR specimens (waterproofed with aluminium tape) of all six labs with the linear regression line. Error bars indicate the standard deviation.

After oven drying and waterproofing with aluminium tape, the specimens were brought in contact with water to perform the capillary water absorption test. They were taken out after specific time intervals, the excess water was wiped from the surface and the mass of the specimens was determined. The limited size of the specimens allowed for an easy and manageable handling of the specimens, as opposed to larger and heavier concrete specimens reported in literature [49]. Figure 8 shows the average cumulative mass gain plotted versus the square root of time for the specimens of all six labs. In the case of the cracked series (REF and CAPS), the average was calculated using the results of 8–12 specimens, see Table 5. For the uncracked series (UNCR) the results of three specimens were used. The water ingress in the uncracked specimens of lab 5 might have been slightly different as these specimens were not provided with a notch. For lab 1 the exposed area with a width of 14 mm centred around the crack was also enforced on the sides of the specimens and not only on the bottom as was the case for the other labs, except lab 4 for which this was also not done. For lab 4 the exposed area was equal to the width of the notch (on average 4 mm), both on the bottom and side surfaces. Without the lateral waterproofing, water will also have entered into the concrete from the sides, but this effect will not have been dominant since lab 1, 2 and 6 show a similar behaviour.

Van Belleghem et al. [50], who did a comparison of an experimental study and a finite element analysis, highlighted that it is the geometry and the distribution of the concrete zones in contact with water which is determinative for the capillary water uptake and not only the surface area exposed to water. They concluded that for an uncracked cementitious matrix, the water ingress will follow a unidirectional mode in the case of no waterproofing, while the ingress will follow a multidirectional mode in the case of partial waterproofing. Thus, the fact that a partial waterproofing was applied is more important than the actual dimensions of the concrete zone in contact with water. This statement is underlined by Table 7, which shows the initial rate of water absorption *I* (in mm/√s) from 10 min of water contact to 6 hours of water contact. This initial rate of water absorption was determined based on ASTM C1585 by normalising the linear slope of the mass gain (in g) versus the square root of time (in √s) by the exposed bottom area of the specimen (in mm^2^) and the density of water (in g/mm^3^). Due to the much narrower exposed area of the specimens of lab 4, *I* is much larger than measured by the other labs, e.g. *I* measured by lab 4 is approximately 10 times higher than *I* measured by lab 1, while the mass gain is only 2 to 3 times as high (see Figure 8). Therefore, it must be concluded that normalising the mass gain by the exposed area is only useful when the exposed area and the specimen size are exactly the same for all specimens. Additionally, it is highlighted that, aside from not waterproofing the exposed bottom surface of the specimens, the specimens in ASTM C1585 are uncracked. As a result, the water ingress is unidirectional. In the current study, the specimens were cracked and partially waterproofed, both of which contribute to a multidirectional ingress. Therefore, the subsequent results are not normalised by the exposed surface area.

**Table 7.** Mean initial rate of water absorption *I* (in mm/√s, determined based on ASTM C1585) of the REF, CAPS, and UNCR specimens of the six labs.

	REF	CAPS	UNCR
Lab 1	0.089	0.089	0.031
Lab 2	0.100	0.097	0.039
Lab 3	0.238	0.167	0.164
Lab 4	0.843	0.733	0.466
Lab 5	0.187	0.158	0.112
Lab 6	0.113	0.120	0.051

Lab 3, 4 and 5 obtain a higher cumulative mass gain than labs 1, 2 and 6. Additionally, the variation on the CAPS results of labs 3 and 4 is also significantly higher, as indicated by the error bars which represent the standard deviation on the mean. Somewhat surprising is that the variation on the uncracked specimens of lab 3, 4, 5 and 6 is significantly higher than the variation measured by lab 1 and 2. There are four different explanations for these sets of observations. The first being the variation on the outflow of the healing agent, which might explain part of the variation on the CAPS series of lab 3 and 4. Depending on the outflow of the PU in the crack – although this seems to have been limited for lab 1, 2 and 6 – some specimens had a lower water uptake. It should be noted that for some specimens the reverse was also observed. For example, the cumulative mass increase after 24 hours of one of the CAPS specimens of lab 4 was not only significantly higher than the other CAPS specimens but was also 16% higher than the highest mass increase of the REF specimens. The second explanation accounts for the behaviour of cracked specimens in general. Looking at Table 6 and Figure 7 it can be seen that the variation on the crack width of some series was rather large. It has been reported in literature that there is a linear relationship between the crack width and the sorption coefficient, although it has been indicated that the goodness of fit (R^2^ ≈ 62%) is not very high as a result of the crack tortuosity which also has an influence [26]. The third and fourth explanations are operator sensitivity and imperfect waterproofing (e.g. caused by deficient adherence or creases in the aluminium tape). They might explain why the variation on the results for uncracked samples of lab 3, 4, 5 and 6 is more pronounced then the variation measured by lab 1 and 2. Operator sensitivity plays a role in all tests. Here specifically it might have manifested in the following aspects: the degree of moistness of the cloth to remove the excess water on the surface of the specimens prior to weighing, water which remained on the surface of the specimens or inside the notch during weighing as a result of a too fast execution, the height of the water level during the test, the frequency of water addition in the reservoir to compensate for the absorbed water, etc. This operator sensitivity can of course be expanded with random errors (e.g. differences in the height of the notches), systematic errors (e.g. accuracy of the scales) and different environmental factors (e.g. differences in temperature and relative humidity during the test or during oven drying). Some labs who removed the aluminium tape immediately after testing noted that the concrete was moist in certain areas away from the crack, such as at the edges of the specimens. Additionally, it was also reported that there was capillary water uptake between two layers of tape, depending on how the aluminium tape was folded at the edges.

**Figure 9.**
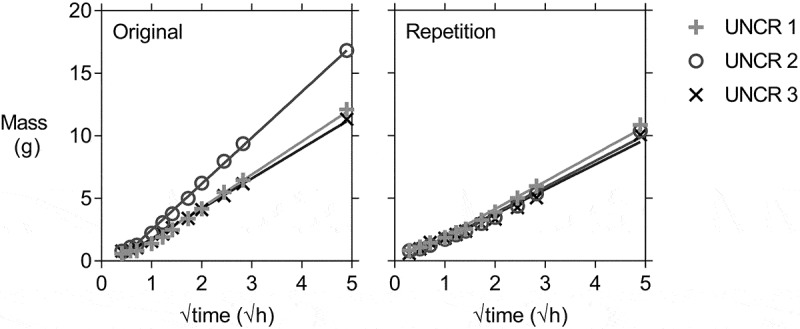
Comparison of the cumulative mass gain versus the square root of time for the uncracked specimens of lab 6 with the individual linear regression lines, showing a more uniform behaviour for the repeated waterproofing with aluminium tape compared to the original waterproofing with aluminium tape.

The effect of the imperfect waterproofing was investigated by lab 6. This lab chose at random half of its REF and CAPS specimens, as well as all three of their UNCR specimens, and removed the aluminium tape. They took extra care in waterproofing the specimens again with aluminium tape, paying special attention to the folds at the edges and tried to prevent any creases and bubbles in the taped area. These specimens were dried again (prior to the application of the new aluminium tape waterproofing) and the capillary water absorption test was repeated. The number of specimens on which this repeated capillary water absorption test with aluminium tape waterproofing was executed is shown in Table 5. Figure 9 shows the comparison of the cumulative mass gain over time of the uncracked specimens with the original waterproofing and the mass gain obtained in the repeated test with the new waterproofing. In the original test, one of the specimens clearly had a deviating behaviour as the water uptake was on average more than 40% higher than for its companion specimens. In the repetition, the results of all three specimens were much more consistent. This was supported by analysis of the equality of the slope of the linear regression curves (level of significance = 5.0%), which showed no equality for the original test (*p* < 0.1%) but revealed equality for the repeated test (*p* = 18.7%), thus highlighting the importance of the quality of execution of the waterproofing. Based on these results, all labs (except lab 3) removed the aluminium tape from the specimens and applied a water impermeable coating to investigate the influence of the waterproofing more extensively (see section 2.2.3 for the exact preconditioning). The labs waterproofed the same zones as they did with the aluminium tape, but left also a zone on the sides (centred on the crack) free of coating to make sure that no coating would enter into the crack. Table 5 shows the number of specimens each lab tested.

In order to calculate the sealing efficiency according to equation 1, the slope of the linear regression curve was determined both for waterproofing with aluminium tape and again for waterproofing with coating. It was noted that for nearly all cracked specimens the value after 24 hours of water contact fell below the regression curve, which was also the case when the specimens were waterproofed with aluminium tape (see Figure 8). In contrast, the uncracked specimens showed an opposite behaviour; the measurement after 24 hours was slightly above the linear regression curve (except for the specimens of lab 3). Upon a closer examination of the results for waterproofing with aluminium tape and also for waterproofing with coating, it appeared that there was a transition around 6 hours for the cracked specimens at which the slope slightly decreased. This might have been the result of saturation of the concrete at the top of the specimens. When cracked specimens are placed in contact with water, the cracks fill up with water almost instantly, as has been studied by neutron imaging and X-ray radiography on mortar samples [27,50,51]. This allows for a fast transport of water to the concrete above the crack tip. The amount of concrete at the tip of the crack (i.e. at the top of the specimen) is much more limited than the amount of concrete at the crack walls and near the crack mouth, meaning that it will be saturated faster, after which the driving absorption force will decrease. The transition was taken into account by determining two sorption coefficients in addition to the standard sorption coefficient prescribed by EN 13057 from 10 min of water contact to 24 hours of water contact (SC_0-24_). The first additional sorption coefficient was determined from 10 min of water contact to 6 hours of water contact (SC_0-6_), and the second sorption coefficient was determined from 6 hours of water contact to 24 hours of water contact (SC_6-24_). The determination of a sorption coefficient up to 6 hours is also prescribed by ASTM C1585 for uncracked concrete (as mentioned previously), although this standard requires the second sorption coefficient to be determined between 1 day and 8 days. Recent research has highlighted that the water uptake in an uncracked cementitious matrix shows a more linear behaviour when plotted against the fourth root of time [52,53]. However, this did not give a satisfactory result for the cracked specimens. For the sake of consistency, the sorption coefficient of the uncracked specimens was calculated with regard to the square root of time.

**Figure 10.**
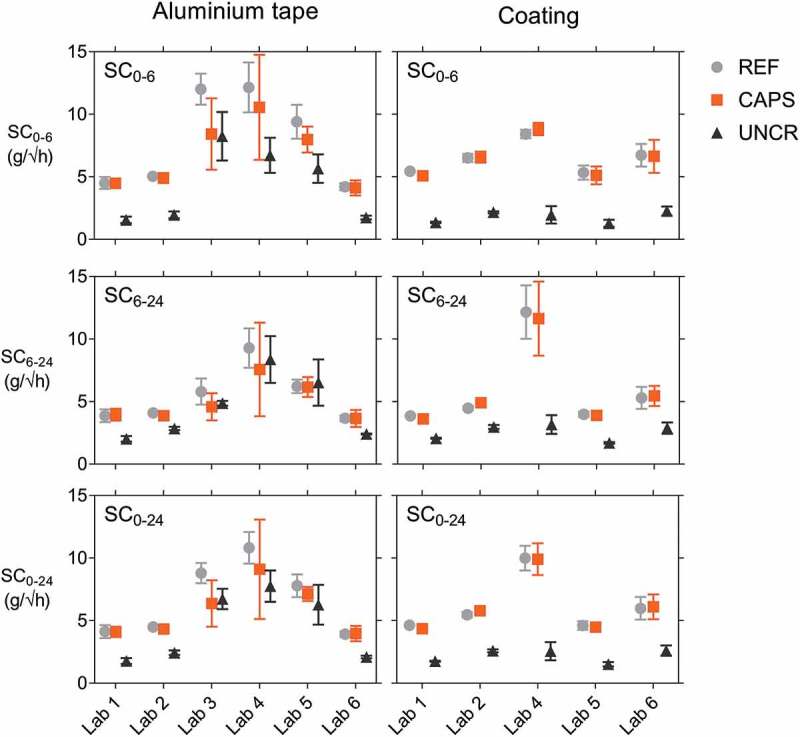
Sorption coefficients SC_0-6_, SC_6-24_ and SC_0-24_ of REF, CAPS and UNCR specimens highlighting that an improved waterproofing quality results in more homogenous results. Error bars indicate the standard deviation.

Figure 10 gives a comparison of the three sorption coefficients, SC_0-6_, SC_6-24_ and SC_0-24_, in the case of waterproofing with aluminium tape and in the case of coating. The results with aluminium tape for lab 6 are the ones from the repeated test after removing and applying new aluminium tape. For the cracked specimens (REF and CAPS) with aluminium tape, it was clear that SC_6-24_ is lower than SC_0-6_. As explained before, this was a result of the saturation of the concrete above the crack tip. All sets of specimens tested by the different labs exhibited this behaviour, but it was distinctly visible for labs 3, 4 and 5. On the other hand, the sorption coefficient of the second period SC_6-24_ of the uncracked specimens is higher than the one of the initial period SC_0-6_, as can, for example be seen for the uncracked specimens of lab 5 with aluminium tape which gave a SC_6-24_ equal to that of the cracked specimens. While this was true for all labs, the large difference observed in lab 5 could have originated from an imperfect sealing of one of the specimens which led to an increase of the SC. The difference is less pronounced for the coated repetition. The uncracked specimens with aluminium tape of lab 3 were an exception to this observation that SC_6-24_ is higher than SC_0-6_. Many of these specimens, also the cracked ones, had a jump in their cumulative mass gain between 1 h 30 min and 2 h. This was the result of excessive water addition in the reservoir to compensate for the drop in water level due to absorption and evaporation. Overall, looking at the results with aluminium tape, the same conclusions can be drawn as for the results in Figure 8: lab 1, 2 and 6 obtained similar results, the results of lab 3, 4 and 5 were significantly higher and also had a higher variation, which in some cases was not explained by a higher mean value.

The results with coating were distinctly more consistent (Figure 10), highlighting the influence of the quality of the waterproofing and potentially also the training of the operator. For example, for lab 4 and lab 5 the CoV on SC_0-6_ of the REF specimens dropped from 16.4%, respectively 14.4%, for waterproofing with aluminium tape to 4.0%, respectively 10.7%, for waterproofing with coating. Another example is the average value of SC_0-24_ of the UNCR specimens which, depending on the lab, varied from 1.78 (lab 1) to 7.76 g/√h (lab 4) for waterproofing with aluminium tape, while it varied from 1.52 (lab 5) to 2.61 g/√h (lab 6) for waterproofing with coating. Comparing the results of cracked specimens with coating, lab 4 obtained a higher SC_6-24_ and SC_0-24_. Many specimens of lab 4 had a cumulative mass gain at 24 hours which was above the linear regression curve. Possibly, too much water was added after the measurement at 8 hours to make sure that the water level was high enough overnight, resulting in an extra water uptake between 8 and 24 hours. The variation on the results with coating of lab 6, especially SC_0-6_, was slightly higher than for the results with aluminium tape. The specimens of lab 6 were coated twice, but still there were some small holes in the coating as a result of the high viscosity of the coating and entrained air from mixing the two components of the coating, possibly explaining the slightly higher variation. This demonstrates that also when choosing for waterproofing with coating the application has to be executed meticulously. It is important to realise that even with a perfect waterproofing and (almost) identical crack widths there will still be a non-negligible variation on the results as a consequence of differences in internal crack geometry, which will change over time due to swelling of the cementitious matrix.

**Figure 11.**
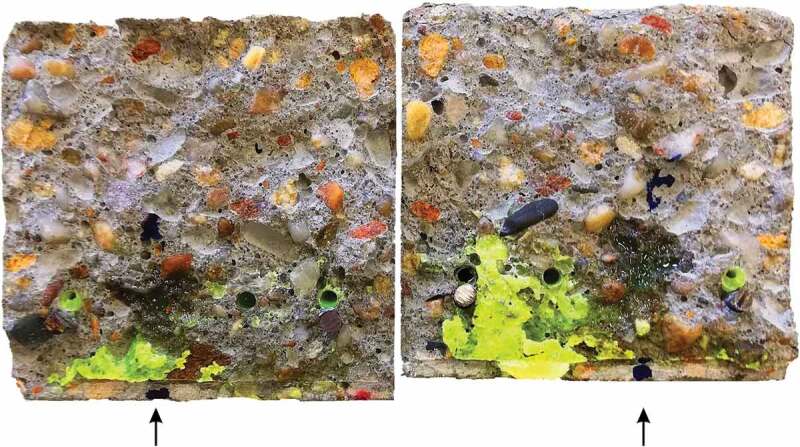
Crack surfaces (60 x 60 mm^2^) of one of the best performing CAPS specimen of lab 1. The hardened PU can be seen as fluorescent yellow. Arrows indicate the capsule from which fresh PU (visible as a dark stain) leaked on the surface when the specimen was broken. Image is highly saturated to improve visibility.

The sealing efficiency SE was calculated for each lab three times using either SC_0-6_, SC_6-24_ or SC_0-24_, all determined from the results of the coated samples. All labs obtained a nearly negligible sealing efficiency (<14%). To investigate the poor sealing efficiency, some specimens with capsules were opened completely. Figure 11 shows the crack surfaces of one of the best performing specimens of lab 1. The PU which was hardened and present in the crack at the moment of capillary water absorption testing is clearly visible as fluorescent yellow. The PU outflow from some of the capsules appears to have been limited, explaining the low sealing efficiency. For the displayed specimen the crack mouth was not completely filled with PU. When splitting the specimens to investigate the crack surfaces, some fresh PU leaked onto the crack walls, seen as dark stains on Figure 11. This indicates that after cracking a minor amount of PU leaked into the crack and polymerised, thereby resealing the broken capsules and preventing the slightly reacted PU inside the capsules from polymerising completely. When splitting the specimens, this PU plug failed. The PU that was still in the capsules was pushed out as a result of the trapped CO_2_ inside the capsule, formed by the initial incomplete polymerisation reaction. The sealing of the capsules used in this study was done using epoxy, instead of methyl methacrylate as was done in previous studies [25,26,28,30,32]. A comparison of these two techniques pointed out that the PU inside capsules sealed with methyl methacrylate undergoes to some extent an initial polymerisation reaction prior to cracking which pressurizes the capsules with CO_2_. As a consequence, the PU is pushed out at the moment that the capsule breaks. In the case of capsules sealed with epoxy, the rate of the initial polymerisation reaction is significantly lower. This might be the result of a better sealing of the capsule, since epoxy is less permeable to gas and moisture than methyl methacrylate (although it should be noted that the permeability is amongst others also dependent on the thickness). Consequently, the PU inside the capsules sealed with epoxy in this study was not pressurised at the moment of cracking the specimens, explaining the low outflow of PU in the cracks.

### 3.2. Mortar specimens

#### 3.2.1. Fresh and hardened properties of mortar

For each lab a separate batch of mortar was prepared at Ghent University. For each individual batch the workability (by flow table test), fresh density and air content were determined, see Table 8. The workability of all batches was comparable with the exception of the batch for lab 3 for which the workability was a little higher. The air content varied between 3.0% and 5.4%.

**Table 8.** Fresh (flow, fresh density, air content) and hardened properties (hardened density, bending strength, compressive strength at 14 days of age) of the different mortar batches used to cast the specimens of each lab (*µ* = mean, CoV = coefficient of variation, NA = not available).

				Hardened density (kg/m^3^)		Bending strength (MPa)		Compressive strength (MPa)	
	Flow (cm)	Fresh density (kg/m^3^)	Air content (%)	*μ*	CoV	*μ*	CoV	*μ*	CoV
Lab 1	20.0	2201	5.4	2246	0.8%	6.91	5.8%	46.6	1.7%
Lab 2	19.8	2205	4.1	2263	0.1%	7.38	1.6%	51.1	4.5%
Lab 3	23.0	NA	3.0	2269	0.2%	6.62	2.3%	53.1	1.8%
Lab 4	19.8	2169	5.4	2227	0.7%	6.84	7.6%	47.8	3.0%
Lab 5	NA	NA	NA	2229	0.1%	5.99	5.8%	46.7	3.2%
Lab 6	19.0	2168	4.4	2235	0.2%	7.12	3.2%	49.5	4.6%

The hardened density was determined at the moment of strength testing at an age of 14 days. The average compressive strength, which was measured by Ghent University, varied between 46.3 MPa and 53.1 MPa. It is noted that the maximum compressive strength was measured for the batch of mortar having the largest workability and the lowest air content.

#### 3.2.2. Crack width of mortar specimens

**Figure 12.**
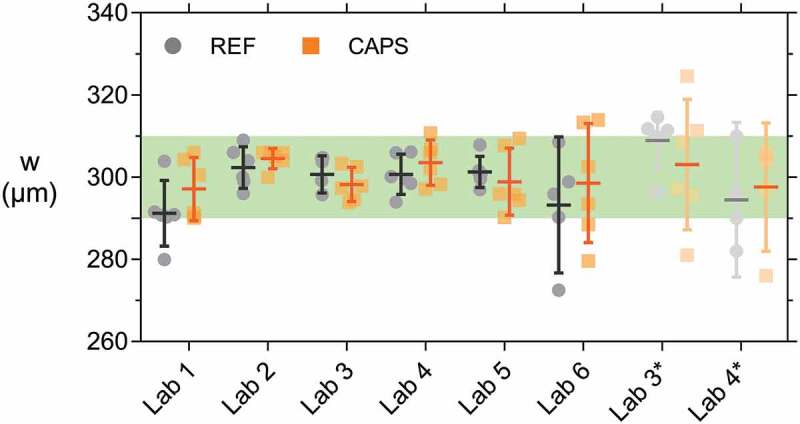
Crack width *w* of individual mortar specimens for which the mean of the series is indicated by a horizontal line and error bars give the 95% confidence interval on this mean. Most mortar specimens had a crack width *w* within the desired crack width range (shaded area).

After cracking the mortar specimens, they were restrained using screw jacks. The final mean crack width *w* measured by all labs after the active crack width restraining process is given in Figure 12. This figure shows the individual values, as well as the mean crack width of the series (horizontal lines) and the 95% confidence interval on this mean (error bars) for both REF and CAPS specimens. The green shaded band indicates the desired crack width range (290–310 µm). Lab 3 and lab 4 did a pre-test on a separately sent batch of specimens to familiarize themselves with the test technique, these are denoted by lab 3* and lab 4*. The crack width of many of these specimens fell outside of the intended range. Additionally, the screw jacks were not applying a significant pressure on the specimens, because the crack width of these specimens was rather limited after crack creation by three-point bending. In the repeated test the crack was opened further so that the screw jacks were fulfilling their intended use of pushing the two halves of the specimens back together. As a consequence of the higher variation on the crack width in the pre-test, the water flow results were also inconsistent (see section 3.2.3). On top of this, lab 4* only had four instead of six6 REF specimens due to damage of one specimen during shipping and the uncharacteristic eccentric cracking of another specimen. Therefore, the results of lab 3* and 4* are represented here only for the sake of completeness and will not be taken into account in the further analysis.

The variation on the crack width of the mortar specimens is undoubtedly lower than the one of the concrete specimens. Overall, the application of the active crack width control technique using the screw jacks was successful for obtaining crack widths within the desired crack width range of 290–310 µm; most of the individual crack widths of the specimens of labs 1–6 fell within the prescribed boundaries. When looking at all 71 specimens (each lab tested six REF and six CAPS specimens, except for lab 6 for which one REF specimen was lost in preparation) only 4 specimens had a crack width smaller than 290 µm, and only 3 had a crack width larger than 310 µm. For 1 out of the 4 specimens with a crack width smaller than 290 µm and all 3 specimens with a crack width larger than 310 µm the deviation was smaller than 4 µm. It is noted that the boundaries of the desired crack width range which were used in this study are only 20 µm, which is significantly lower than in previous studies on similar samples which employed a passive crack width control using tensile reinforcement and a displacement-controlled loading system. These studies reported ranges of 40 µm [45] to 50 µm [44].

The crack width of the REF samples was equal to the CAPS specimens within each lab, as verified by independent sample t-tests (level of significance = 5.0%, all *p*-values >20.0%). Based on this, the REF and CAPS values were combined to study if there was a significant difference for the crack widths obtained by the different labs. Equal variances could not be assumed (level of significance = 5.0%, *p* = 0.1%). According to a Welch test and a Brown–Forsythe test not all means were equal (level of significance = 5.0%, *p_Wel__ch_* = 1.7%, *p_Brown-Forsythe_* = 4.1%). A subsequent Tamhane’s T2 post hoc test showed that only lab 1 and lab 2 had a slightly different mean crack width (level of significance = 5.0%, *p* = 2.8%).

To conclude, the active crack width control technique resulted in most specimens having a crack width which fell within the desired crack width range. As a consequence, similar results were obtained between the REF and CAPS specimens within each lab, and all labs obtained (nearly) comparable results.

#### 3.2.3. Water permeability of mortar specimens

**Figure 13.**
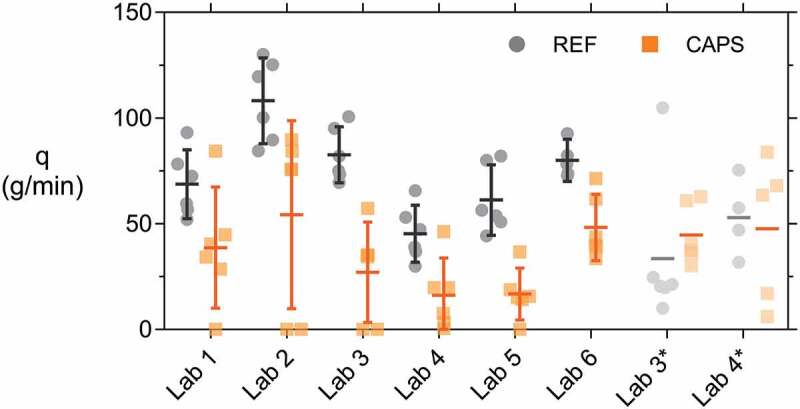
Individual flow rates *q* and the means of the different specimen batches indicated by horizontal lines. Error bars give the 95% confidence interval on the mean.

Following the active crack control of the specimens and the subsequent submersion, the specimens were subjected to a water flow test (see section 2.3.3). Figure 13 shows the water flow *q* (g/min) leaking from the samples during the test. The results of lab 3* and 4* were inconsistent with the rest of the results, as already mentioned in section 3.2.2. For example, for lab 3* one of the REF specimens obtained a flow more than 4 times higher than the second largest flow within that series. For lab 4* the coefficient of variation on the REF values was higher than for any other lab (excluding lab 3*; COV_lab4*_ = 34.5%). Additionally, the CAPS flow value of lab 3* and 4* was uncharacteristically high in comparison with the REF flow value – for lab 3 the flow was even higher for the CAPS specimens than for the REF specimens. These inconsistent results can be explained by the high variation on the crack widths and the fact that the screw jacks were not actively controlling the crack due to a limited crack opening when the specimens were removed from of the loading setup. Similar as for section 3.2.2, these results will not be taken into account in the further discussion.

The variation on the water flow was significantly higher than for the crack width, see Table 9. It can be reasoned that for the CAPS specimens a high variation can be expected, as the flow of these specimens is dependent on the outflow of PU in the crack. However, for the REF specimens the variation on the water flow is also larger than the variation on the crack width. The crack width was only measured manually at discrete points at the surface of the specimen. Even though guidelines were provided to help select these points in a consistent way for the different labs (see supplementary material), it is possible that these discrete points were not entirely representative of the crack itself or that there were local defects (e.g. missing aggregates) which had a dominant influence on the water flow. Additionally, the flow is also influenced by the internal crack geometry or tortuosity. Even for low variations on the crack width, it has been reported that the variation on the flow can be a magnitude higher as a result of differences in crack tortuosity [33]. This crack tortuosity cannot be controlled in a mechanical cracking process. Techniques such as tomography could allow to study the interior of these cracks, but they are often expensive and time-consuming.

**Table 9.** Mean µ and coefficient of variation CoV for the measured crack width *w* and water flow *q* for both REF and CAPS specimens of the six labs.

	REF				CAPS			
	*w*		*q*		*w*		*q*	
	*µ* (µm)	CoV	*µ* (g/min)	CoV	*µ* (µm)	CoV	*µ* (g/min)	CoV
Lab 1	291	2.6%	68.7	22.6%	297	2.5%	38.7	70.6%
Lab 2	302	1.6%	108.2	17.9%	305	0.8%	54.3	78.1%
Lab 3	301	1.4%	82.6	15.3%	298	1.3%	27.0	83.8%
Lab 4	301	1.6%	45.3	28.4%	304	1.7%	16.2	104.0%
Lab 5	301	1.2%	61.2	25.9%	299	2.6%	16.7	70.2%
Lab 6	293	4.5%	79.9	10.1%	299	4.6%	48.2	30.9%

Comparing the results of the REF specimens to the CAPS specimens in Figure 13, it is evident that the variation is much higher for the CAPS specimens. This is of course to be expected since there is an extra factor which contributes to variability, namely the spread of the PU healing agent or in other words the degree of self-healing. For example, the 95% confidence interval on the mean of the CAPS specimens of lab 2 is the largest of all groups, even though four out of the six specimens have a very similar water flow of 80 g/min. However, the other 2 specimens have a significantly different water flow; their cracks are perfectly sealed and as a consequence their water flow is equal to 0 g/min, explaining the wide confidence interval. Therefore, it is most accurate to compare the results of the REF specimens to investigate the accuracy of the water flow test. A Levene’s test based on the mean indicated that equal variance could be assumed (level of significance = 5.0%, *p* = 13.0%). A one-way ANOVA test (one-way analysis of variance) indicated that there was a difference in the means of the REF series over the different labs (level of significance = 5.0%, *p* ≈ 0%). A subsequent Tukey’s HSD (Honestly Significant Difference) post hoc test (level of significance = 5.0%) identified 3 groups: group 1 consisted of lab 1, 4 and 5 (*p_min_* = 9.1%), group 2 consisted of labs 1, 3, 5 and 6 (*p_min_* = 14.8%), and group 3 consisted of lab 2 and 3 (*p* = 5.2%). To account for unequal sample sizes (lab 6 had only five REF samples instead of six) also a Hochberg’s GT2 and Gabriel’s post hoc test were executed. They identified the same three groups as the Tukey test and highlighted that the results of lab 3 and 6 are almost equal to one another (*p_Gabriel_* = 4.7%, *p_Hochberg_* = 4.8%). Despite the fact that there was some difference between the groups, it is positive to see that none of the labs obtained an excessively different result from the others.

Looking at the results of the REF specimens in Figure 13, the same conclusions can be drawn as for the statistical analysis. Lab 2 has the highest flow rate and its confidence interval overlaps with that of labs 3 and 6. Lab 4 has the lowest flow rate and has overlap with labs 1 and 5. It should be noted that, unlike all other labs, lab 4 did not keep the water head constant. As a result, the driving force decreased over time. For the specimen with the largest flow, the water drop at the end of the test was about 14% with respect to the prescribed water head of 50 cm. This partly explains the somewhat lower results of lab 4. Even though not all labs obtained a (REF) flow which was statistically equal, the results have the same order of magnitude and are as such comparable, which is a significant improvement compared to a previous Round Robin Test [57,44].

**Figure 14.**
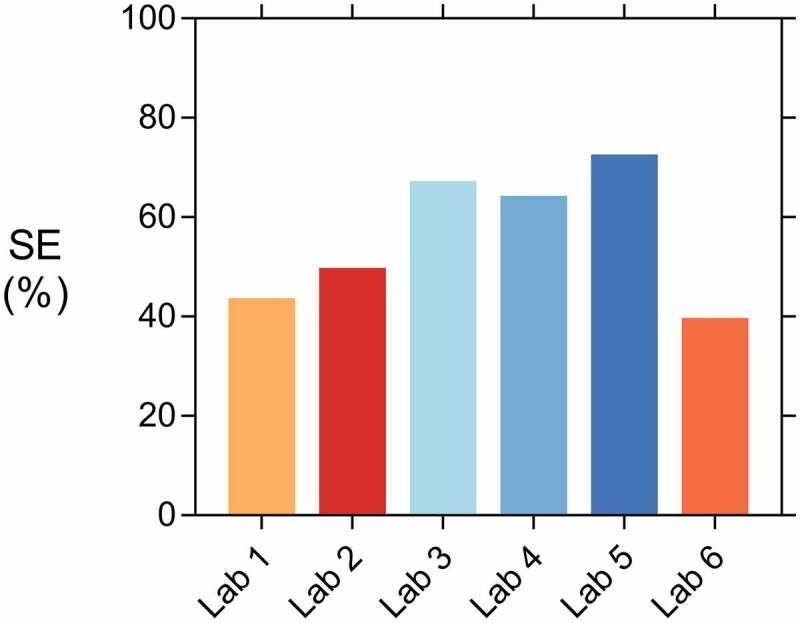
The sealing efficiency SE_flow_ measured in the different labs.

**Figure 15.**
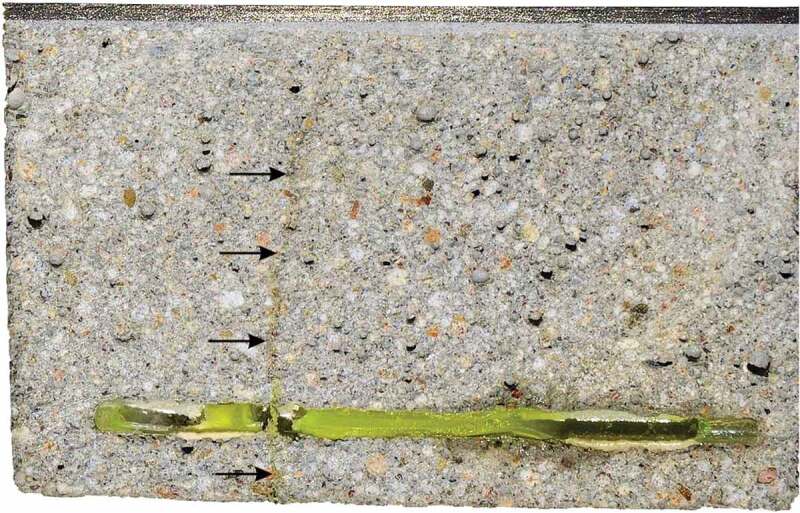
Mortar specimen with mortar removed from the side up to the location of a capsule (length approximately 55 mm) showing that most of the PU was retained in the capsule. Arrows indicate the location of the crack.

In the end, the most interesting result is of course the obtained sealing efficiency. Figure 14 shows the sealing efficiency *SE* of all labs, calculated according to equation 2. It can be seen that the result of lab 2 now fall in line with the other labs; the high flows for both REF and CAPS specimens level each other out. A similar conclusion can be drawn for lab 4. The sealing efficiency of all labs varied from 40% to 73%. Looking at Figure 14, the sealing efficiencies can be divided in two groups: one group with an efficiency varying from 40% to 50% and another group with an efficiency varying from 64% to 73%. Yet, overall the results of all labs point in the same direction; the sealing efficiency is promising but definitely not perfect. This imperfect sealing of the cracks has the same reason as for the concrete specimens: an insufficient leakage of PU into the crack. This is demonstrated by Figure 15 which shows a mortar specimen with the mortar removed up to the position of a capsule. It is clear that only part of the PU leaked into the crack, the other part polymerised inside of the capsule.

It should be emphasized that for most operators in the different labs this was the first time to work with this kind of healing technique and this kind of test method. It is expected that familiarity with the technique would harmonize the results. This study focused on self-healing mortar with macrocapsules, but the combination of the water flow test in combination with an active crack width control technique can easily be applied for other healing techniques. Due to the fast execution time, it has, for example already been applied to study the sealing efficiency of superabsorbent polymers [54] and superabsorbent polymers in combination with nanosilica [55].

#### 3.2.4. Spread of healing agent on the crack surface of mortar specimens

**Figure 16.**
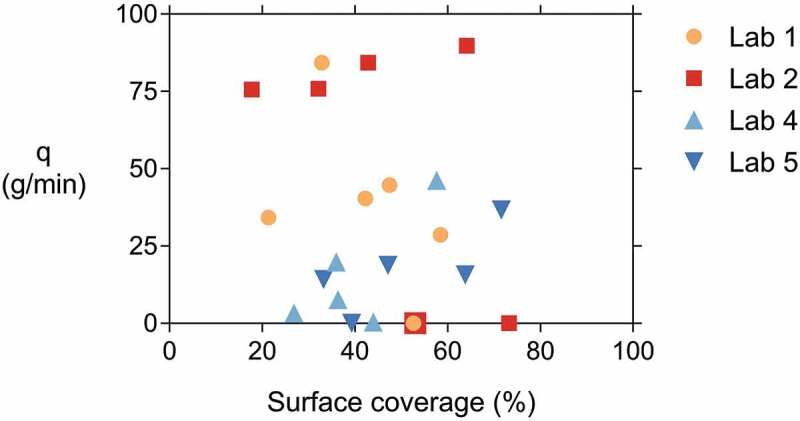
Water flow *q* versus the PU surface coverage for labs 1, 2, 4 and 5.

After performing the water permeability test, labs 1, 2, 4 and 5 split their CAPS specimens open at the location of the crack to analyse the spread of the healing agent. For two of the specimens of lab 4 one of the capsules was in a higher location than its preplacement position, see Figure 3. Most likely this happened during filling and compacting of the moulds. For all the other labs the capsules were in their expected position. The labs noted a similar average surface coverage of: 42.5% (lab 1), 47.20% (lab 2), 40.2% (lab 4), and 51.0% (lab 5). The slightly higher surface coverage obtained by lab 5 could explain the higher sealing efficiency, see Figure 14. Figure 16 shows the water flow of the different specimens in function of their surface coverage. It is noted that it is impossible to obtain a perfect surface coverage since the area of the hole (used to induce the water in the permeability test) was taken into account in the total area. Anglani et al. [37] studied the same PU healing agent but encapsulated in one cementitious capsule with a larger diameter. These capsules had either an internal or an external epoxy coating to make them moisture-proof. They measured an average surface coverage of 36% for specimens with internally coated capsules and 47% for specimens with externally coated capsules. These values are comparable to the average surface coverage of the different labs, although it is noted that the variation is higher for the results reported here. The sealing efficiency measured by Anglani et al. [37], using the same permeability setup as is used in the current study, was equal to 79%, respectively 28%, for the specimens with an internally, respectively externally, coated capsule. Lab 1, 2, 4 and 5 obtained a sealing efficiency between 44% and 73% which falls in between these values.

**Figure 17.**
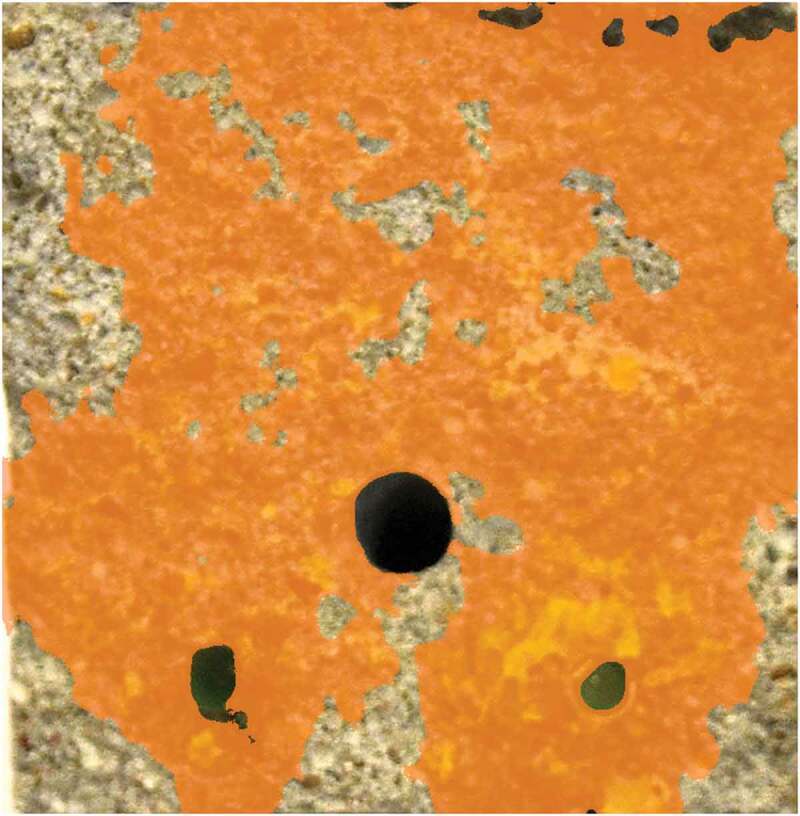
Crack surface (40 x 40 mm^2^) of one of the specimens with the best PU surface coverage but still a significant flow due to an unconnected crack filling below the cast-in hole (through which water was induced during the permeability test). The crack mouth through which water leaked out of the specimen is oriented to the bottom, like in the water flow test.

From Figure 16 it could also be concluded that a high surface coverage does not directly translate into a good sealing efficiency. There seems to be no strong relationship between the surface coverage and the water flow. Indeed, the surface coverage only has an indirect influence on the water flow. Figure 17 shows a crack surface overlaid with its filtered segmented image determined using the Trainable Weka Segmentation plugin in ImageJ, see section 2.3.4. The surface coverage of this sample was one of the highest (71.6%), yet the zone below the hole was not completely covered with PU, leaving a path for the water to leak out of the specimen. To seal a crack, it is sufficient if the crack mouth is filled with healing agent, or in the studied specimens if the water-inducing hole is surrounded with healing agent. For many specimens it was noted that the PU spread regions from the individual capsules did not connect, and thus a barrier to withstand the water pressure could not be created.

The surface coverage which is reported here should be interpreted in a qualitative way. The applied procedure to determine the surface coverage was susceptible to subjective interpretation and errors. Even though a lot of effort was put in standardising the procedure, the machine learning algorithm had to be trained by manually selecting regions with an without PU, which was to some extent susceptible to subjective interpretation. Based on these manually selected regions entire images could be segmented. These segmented images were then manually checked for misidentified zones with the possibility of over- or undercorrecting. For labs 1 and 4 the opening of the specimens caused a leakage of fresh PU onto the cracked surface similar as explained in section 3.1.3. Since the fresh PU covered the hardened PU, it was not possible to discern between the two. The reported surface coverage of these labs might thus be overestimated.

**Figure 18.**
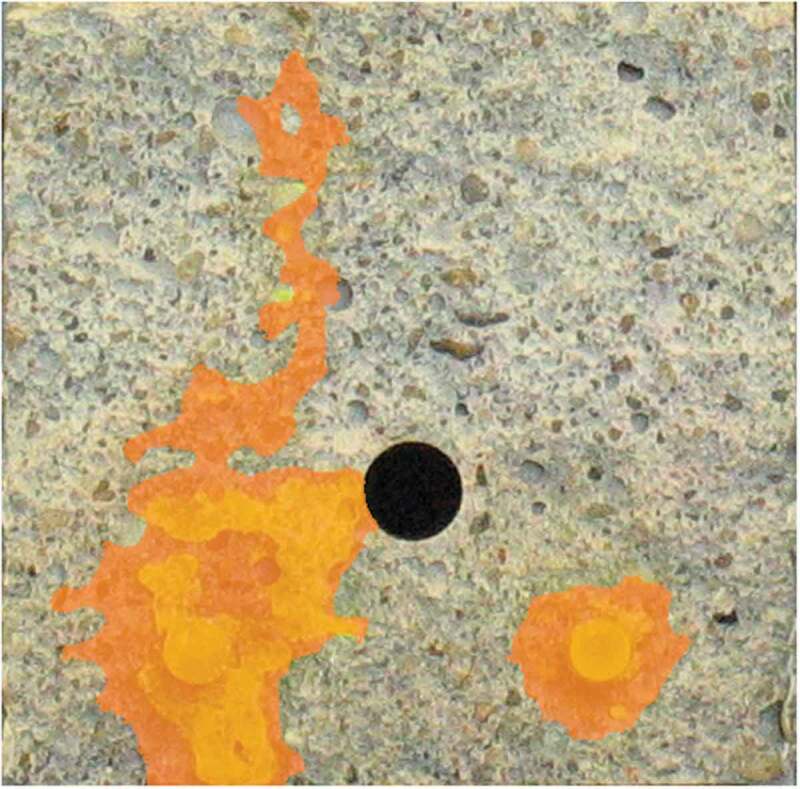
Crack surface (40 x 40 mm^2^) of specimen with limited PU surface coverage. The crack mouth through which water leaked out of the specimen is oriented to the bottom, like in the water flow test.

The specimens were stored with their crack facing downwards when the PU was polymerizing. This is representative for bending cracks in beams and slabs which are loaded by dead weight and downwards acting loads. As mentioned in section 2.3.2, the crack of the mortar prisms was facing upwards for a very short time to allow for the execution of the active crack width control. For the CAPS specimens, this might have influenced the outflow of the PU from the capsules, resulting in some more healing agent flowing towards the crack tip (where the crack is the narrowest). To obtain a good sealing in the water permeability test the healing agent needs to fill the crack mouth (from where water will leak out of the specimen). Thus, the short time that the capsules were turned upwards (favouring a spread of PU towards the crack tip) will not have improved the sealing of the cracks. Additionally, it is noted that the capillary forces are the highest at the crack tip of the specimen, so in any case it is likely for the PU to be pulled into this zone. As an example, Figure 18 shows a crack surface overlaid with its filtered segmented image of a specimen with limited PU surface coverage. It can be seen that only a small amount of PU flowed out of the right capsule and that the outflow was centred around the capsule. For the left capsule, there was a bit more outflow. Some of this outflow is located towards the crack tip, possibly as a result of the crack orientation during crack width control, but it is evident that the outflow above the cast-in hole did not influence the obtained sealing efficiency.

## 4. Conclusions

In this paper, test techniques suited for assessing the performance of self-healing concrete with macrocapsules were investigated by an inter-laboratory testing program: a capillary water absorption test for concrete specimens and a water permeability test for mortar specimens.

The reinforced concrete specimens were cracked in a three-point bending setup by using a displacement-controlled loading system. The type of system (LVDT, CMOD, or DIC) varied between the different labs, as well as the final crack opening value prior to unloading. Some labs obtained a high variation on their crack width. There was also quite some variation between the different labs, but the labs which opened the cracks to a value as high as 485 µm (either with LVDT or CMOD) were able to obtain the target crack width of 300 µm with good accuracy, which can be explained by a partial crack closure due to elastic regain in the reinforcement upon load removal. After drying of the cracked concrete specimens, two capillary water absorption tests were performed, once with aluminium tape waterproofing and once with coated waterproofing. The results with aluminium tape showed a much larger variability than the results for the coating. This highlighted the importance of the quality of the waterproofing when executing a capillary water absorption test, which is more vital than having exactly the same non-waterproofed area of concrete in contact with water. Despite that a capillary water absorption test is very straightforward, the results can be easily affected by the operator sensitivity, e.g. the frequency and amount of water addition in the reservoir to compensate for the absorbed water. If the quality of the waterproofing is safeguarded and the operator sensitivity is limited (by providing very detailed guidelines), the presented results show that different labs can obtain comparable results for the prescribed test protocol. The limited size of the specimens (60 x 60 × 220 mm^3^) allowed for an easy and manageable handling of the specimens. Due to this small size, the top of the cracked specimens became saturated before the end of the test, resulting in a lower slope of the mass versus square root of time graphs after approximately 6 hours. This was solved by determining two additional sorption coefficients (from 10 min to 6 hours and from 6 hours to 24 hours) aside from the sorption coefficient from 10 min to 24 hours.

The prismatic mortar specimens were not provided with tensile reinforcement, but the prism halves remained connected after cracking due to a CFRP strip glued to the top. Cracks were induced in a three-point bending setup without using a displacement-controlled loading system, instead an active crack width control technique was applied immediately after cracking. The resulting crack widths were nearly all within the narrow desired crack width range (290–310 µm). As such, the crack widths of the mortar specimens were much more consistent than for the concrete specimens. Disregarding a minor difference between two labs, the crack widths obtained by the different labs did not show a significant difference. This is paramount for obtaining comparable water permeability results, as these types of tests are extremely sensitive to the crack width, which was highlighted by the result of a pre-test executed by two labs. Despite the fact that two out of the six labs did not obtain statistically equal water permeability values for the REF specimens, the magnitude of the results of all six labs was comparable. When taking into account both the results of the REF and CAPS specimens in the calculation of the sealing efficiency, the two labs who obtained diverging results for the REF specimens obtained analogous results to the other labs. This confirms the potential of the investigated water flow test in combination with an active crack width control technique as a standardized test method. This test method can also be used to assess other self-healing strategies; it has, for example already been used to study the sealing efficiency of superabsorbent polymers and of superabsorbent polymers in combination with nanosilica. An analysis of the spread of the healing agent on the crack surfaces showed no strong relationship between the surface coverage and the water flow. After all, a high surface coverage does not guarantee a perfect crack sealing as long as a water barrier has not been formed.

The healing which was obtained by addition of the glass macrocapsules filled with PU was poor for the concrete specimens (<14%) and moderate for the mortar specimens (sealing efficiency of 40% to 74%). This is the result of a limited outflow of PU from the capsules into the crack. In previous studies, there was a small amount of polymerisation inside the capsules (prior to cracking) triggered by a small amount of moisture diffusion through the methyl methacrylate sealing the tubular glass capsules. During the limited initial polymerisation reaction CO_2_ was produced which pressurized the capsules. This pressurization facilitated the outflow when the capsules broke. In the current study, the sealing of the glass tubular capsules was done with a less permeable material (epoxy instead of methyl methacrylate). Therefore, the initial polymerisation reaction prior to cracking was much more limited. As a consequence, the capsules were not pressurized, and the outflow of PU was reduced.
